# Intraoperative cone-beam CT for real-time quality control in CSF shunt surgery: a prospective workflow study

**DOI:** 10.1016/j.bas.2026.106269

**Published:** 2026-08-18

**Authors:** Lina-Elisabeth Qasem, Dilara Karakaya, Christian Seemann, Nadine Angelika Döpke, Leonhard Mann, Fee Keil, Fatma Kilinç, Paul Kendlbacher, Marcus Czabanka, Vincent Prinz

**Affiliations:** a Goethe University, University Hospital Frankfurt am Main, Department of Neurosurgery, Frankfurt am Main, Germany; b Goethe University, University Hospital Frankfurt am Main, Department of Neuroradiology, Frankfurt am Main, Germany

**Keywords:** Cerebrospinal fluid shunt, Cone-beam computed tomography, Intraoperative imaging, Quality control, Radiation exposure

## Abstract

**Introduction:**

Postoperative CT is routinely performed after cerebrospinal fluid (CSF) shunt implantation to verify catheter position. Intraoperative cone-beam CT (iCBCT) enables real-time verification and may obviate routine postoperative CT.

**Research question:**

Does an iCBCT-based workflow reduce radiation exposure and permit omission of routine postoperative CT?

**Methods:**

This interim analysis of an ongoing, prospective, observational study enrolled 10 patients undergoing ventriculoperitoneal (VP), lumboperitoneal (LP), ventriculoatrial (VA), or cyst shunt implantation with iCBCT-based quality control and compared them with a matched retrospective cohort of 10 patients managed by conventional postoperative CT. The primary endpoint was radiation exposure. Secondary endpoints were operative duration and the number of immediate revisions for catheter malposition.

**Results:**

The volumetric dose index was lower in the iCBCT group than in the control group (CBDIw median 9.27 mGy [IQR, 6.5-11.5] vs CTDIvol 31.15 mGy [IQR, 23.5-34.2]; p = 0.002). Intraoperative imaging detected catheter malposition in one VP shunt and enabled immediate revision within the same surgical session. Routine postoperative CT was omitted in all iCBCT patients, without catheter-related complications through the routine 2–3-week postoperative follow-up. OR time was longer in the iCBCT group due to image acquisition without statistical significance (median 142 min [IQR, 114-157] vs 127 min [IQR, 104-140]; p = 0.2948). Surgical time was comparable between groups (median 65.5 min [IQR, 54–80] vs 60 min [IQR, 50–76]; p = 0.590).

**Discussion and conclusion:**

In this interim cohort, iCBCT lowered the radiation dose index and replaced postoperative CT, enabling real-time catheter verification across CSF shunts.

## Highlights

• iCBCT enables real-time catheter verification across CSF diversion procedures.
• Routine postoperative CT can be omitted, potentially reducing cumulative radiation.
• Intraoperative imaging detected malposition, enabling same-session revision.
• Loop-X-based quality control does not prolong surgical time.
• Single workflow covers VP, LP, VA, and cyst shunt implantation.

## 1 Introduction

Cerebrospinal fluid (CSF) shunting remains a cornerstone of the surgical management of hydrocephalus (Khalil et al., 2024). Postoperative computed tomography (CT) is routinely performed after CSF shunt implantation to verify catheter as well as valve position and to detect early procedure-related complications such as hemorrhage or catheter malposition. This applies to ventriculoperitoneal (VP), lumboperitoneal (LP), ventriculoatrial (VA), or cyst shunts and represents established practice in most neurosurgical centers. In conventional workflows, imaging is obtained after completion of surgery, typically on the first postoperative day.

Reported rates of radiologically defined catheter malposition after VP shunt implantation range from approximately 20-40%, with 5-15% of cases requiring revision surgery (Krause et al., 2024; Brenner et al.). Suboptimal ventricular-catheter position has been associated with reduced shunt survival (Dobran et al., 2018; Lind et al., 2009; Stewart et al., 2025; Whitehead et al., 2017). Clinically relevant catheter malposition or migration has been described in approximately 5-10% of VA shunts (Brenner et al.) and 10-25% of LP shunts, depending on the definition applied and the surgical technique used (Foo et al., 2023; Ho et al., 2023; Jusué-Torres et al.). Corrective procedures require a secondary intervention under repeat anesthesia, with associated logistical effort, prolonged hospitalization, and avoidable patient risk.

Intraoperative cone-beam computed tomography (iCBCT)(Hecht et al., 2020; Kendlbacher et al., 2022) enables immediate verification of catheter placement during the ongoing procedure. Real-time quality control permits prompt correction of suboptimal catheter positioning within the same surgical session (Krause et al., 2024). This approach has the potential to avoid secondary surgery and repeat anesthesia, thereby reducing revision rates and obviating routine postoperative CT in selected patients, with corresponding benefits in terms of shorter hospitalization, reduced patient burden, and possibly lower cumulative radiation exposure.

Prior work has established the technical feasibility and accuracy of intraoperative CT-based imaging in neurosurgery (Hecht et al., 2020; Kendlbacher et al., 2022), including CSF diversion surgery (Krause et al., 2024). Systematic evaluations focusing specifically on shunt implantation, however, remain limited. Comparative data assessing iCBCT versus conventional postoperative CT with respect to immediate revision rates, operative efficiency, and radiation exposure are scarce, and evidence addressing the full spectrum of shunting procedures – VP, LP, VA, and cyst shunts – within a unified workflow is particularly sparse.

The present study evaluates an optimized workflow for CSF shunt implantation incorporating iCBCT-based quality control. Patients undergoing VP, LP, VA, or cyst shunt implantation with intraoperative imaging were compared with a retrospective control cohort managed according to conventional postoperative-CT protocols.

## 2 Methods

### 2.1 Study design

**Fig. 1:**
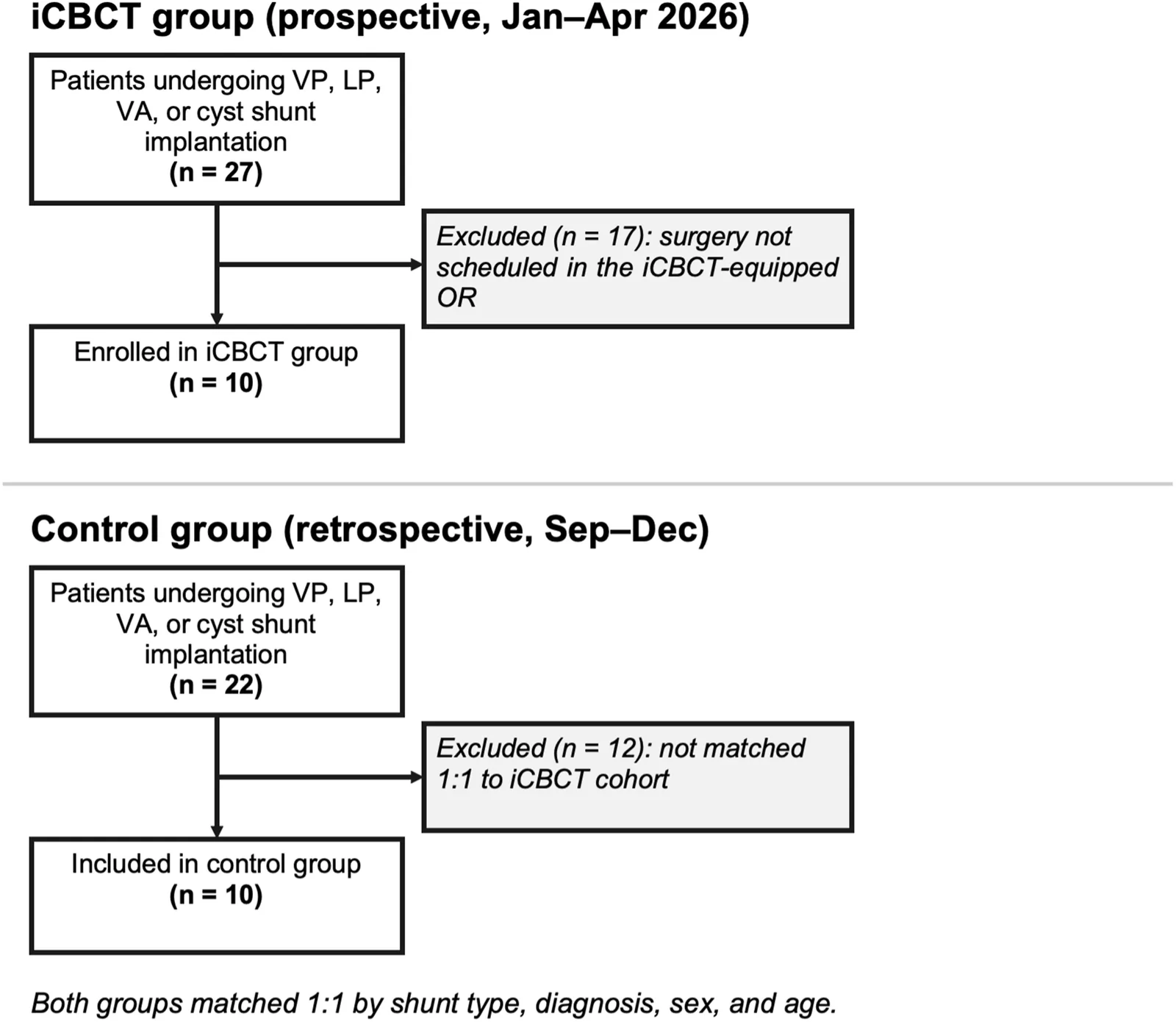
STROBE flow diagram of patient recruitment for the iCBCT group (prospective, January–April 2026) and the control group (retrospective, Septembe–December 2025). Of 27 candidates, 17 were excluded due to OR unavailability, yielding 10 patients in the iCBCT group. Of 22 retrospective candidates, 12 were excluded during 1:1 matching, yielding 10 patients in the control group.

This interim analysis of an ongoing, prospective, single-center observational study enrolled patients undergoing VP, LP, VA, or cyst shunt implantation between January and April 2026 in the iCBCT-equipped operating room (OR), irrespective of the underlying hydrocephalus etiology (iCBCT-group). Recruitment was limited by OR availability rather than by patient eligibility (see STROBE flow diagram, Fig. 1). The retrospective control cohort comprised ten patients undergoing shunt implantation between September and December 2025, matched 1:1 with the prospective cohort by shunt type, diagnosis, sex, and age (control group).

The primary endpoint of this interim analysis of an ongoing registered trial (DRKS00037762) was radiation exposure. Operative duration was a secondary endpoint, defined as surgical time (skin-to-skin) and total OR time. The trial's original primary endpoint (revision rate) will be addressed in a separate publication once recruitment is complete. The present report represents an interim analysis focusing on feasibility and radiation exposure.

### 2.2 Technical note

Intraoperative imaging within the iCBCT group was performed using the Brainlab Loop-X system (Brainlab, Munich, Germany), a mobile robotic cone-beam CT platform integrated into the OR. The system provides 3D volumetric imaging without requiring patient transport.

Imaging was acquired after skin closure with the patient still under general anesthesia. A standardized acquisition protocol was used to obtain volumetric datasets covering the cranial and abdominal regions for VP shunts, the cranial and thoracic regions for VA shunts, and the relevant spinal segments and abdominal region for LP shunts. Datasets were reconstructed immediately and reviewed intraoperatively on a dedicated workstation by the operating neurosurgeon to assess catheter trajectory and tip position. In cases of suboptimal catheter position, immediate revision was performed within the same surgical session, followed by repeat intraoperative imaging to confirm adequate final placement. In the iCBCT group, no routine postoperative CT was performed in the absence of postoperative complications.

In the control cohort, no intraoperative imaging was performed. Postoperative catheter assessment was performed according to institutional practice, typically on the first postoperative day. VP shunts were assessed by cranial CT and abdominal x-ray, VA shunts by cranial CT and thoracic x-ray, and LP shunts by abdominal and spinal CT. Malposition identified on postoperative imaging was managed according to clinical judgment, with secondary revision surgery when indicated.

All patients in both cohorts were seen in our outpatient clinic 2–3 weeks postoperatively as part of routine clinical follow-up, which included CT imaging to assess ventricular width for valve adjustment. This follow-up imaging was also reviewed to exclude late catheter-associated complications.

Radiation exposure for iCBCT and postoperative CT was extracted from system-generated dose reports. No additional imaging beyond routine clinical care was performed for the purpose of the study.

Reporting of radiation dose differs between modalities: diagnostic CT reports the computed tomography dose index (CTDIvol, mGy) and dose-length product (DLP, mGy·cm^2^), whereas CBCT systems and 3D C-arms typically report the dose-area product (DAP, mGy·cm^2^). These metrics are not directly comparable. The Loop-X system additionally calculates a CTDI equivalent for CBCT acquisitions – the weighted cone-beam dose index (CBDIw, mGy) – which enables a standardized estimation of radiation exposure and direct comparison with diagnostic CT. In the present study, CBDIw and CTDIvol were used for volumetric dose comparison. Effective dose (mSv) was calculated from DLP and DAP, respectively, using region-specific conversion factors (k = 0.0021 mSv·mGy^−1^·cm^−1^ for the head; k = 0.015 mSv·mGy^−1^·cm^−1^ for the abdomen for diagnostic CT (American Association of Physicists in Medicine, 2008) and k = 0.065 mSv·mGy^−1^·cm^−2^ for the head (Wang); k = 0.27 mSv·mGy^−1^·cm^−2^ for the abdomen (Hwang et al., 2018) for iCBCT).

### 2.3 Statistical analysis

Data were analyzed using GraphPad Prism (version 9.5.1; GraphPad Software, San Diego, CA, USA). Continuous variables are presented as mean ± standard deviation (SD) or median with interquartile range (IQR), as appropriate. Between-group comparisons were performed using the unpaired *t*-test for normally distributed data and the Mann–Whitney *U* test for nonparametric data. Normality was assessed using the Shapiro-Wilk test. Given the exploratory nature of the study, no formal sample-size calculation was performed. A two-sided p value < 0.05 was considered statistically significant.

### 2.4 Ethics

The study protocol was reviewed and approved by the Ethics Committee of the Faculty of Medicine, Goethe University Frankfurt, Germany (No. 2025-2373) and was conducted in accordance with the Declaration of Helsinki and applicable regulatory requirements. The study was prospectively registered in the German Clinical Trials Register (Deutsches Register Klinischer Studien, DRKS; trial identifier DRKS00037762). Written informed consent was obtained from all patients in the prospective cohort. For the retrospective cohort, the requirement for informed consent was waived by the Ethics Committee of Goethe University Frankfurt am Main, Germany.

## 3 Results

### 3.1 Baseline characteristics

Both groups included six LP shunts, two cyst shunts (cystoperitoneal or cystodural), one VP shunt, and one VA shunt, respectively. The same distribution of underlying diagnoses was observed in both groups: normal pressure hydrocephalus (NPH; n = 5, 50%), tumor-related hydrocephalus (n = 4, 40%), and posthemorrhagic hydrocephalus following subarachnoid hemorrhage (n = 1, 10%). Seven patients in each group were male (70%). Median age was 68 years (IQR, 49–75) in the iCBCT group and 69 years (IQR, 45–71) in the control group. In the iCBCT group, Miethke proGAV valves were used in three cases (the VP shunt and the two cyst shunts), Miethke M.blue valves in the six LP shunts, and the VA shunt was equipped with a proGAV and an M.blue valve in series. In the control group, six patients received an LP shunt with an M.blue valve, and four received a proGAV valve (two cyst shunts, one VP shunt, and one VA shunt). Baseline characteristics are summarized in Table 1.

**Table 1:** Baseline characteristics.

variable	iCBCT group (n = 10)	control group (n = 10)
age (median, IQR in years)	68 (49-75)	69 (45-71)

Sex		
male, n (%)	7 (70%)	7 (70%)
female, n (%)	3 (30%)	3 (30%)

diagnosis		
NPH, n (%)	5 (50%)	5 (50%)
tumor, n (%)	4 (40%)	4 (40%)
posthemorrhagic (SAH), n (%)	1 (10%)	1 (10%)

CSF diversion		
LP shunt, n (%)	6 (60%)	6 (60%)
VP shunt, n (%)	1 (10%)	1 (10%)
VA shunt, n (%)	1 (10%)	1 (10%)
cysto shunt, n (%)	2 (20%)	2 (20%)

valve		
proGAV, n (%)	3 (30%)	4 (40%)
M.Blue, n (%)	6 (60%)	6 (60%)
in series, n (%)	1 (10%)	0

Abbr.: IQR, interquartile range; LP, lumboperitoneal; NPH, normal pressure hydrocephalus; SAH, subarachnoid hemorrhage; VA, ventriculoatrial; VP, ventriculoperitoneal.

*Both groups were matched 1:1 by shunt type, diagnosis, sex, and age.

### 3.2 Radiological and surgical parameters

In the iCBCT group, one intraoperative scan was performed in every patient and a repeat scan in one case after intraoperative revision (median CBDIw: 9.27 mGy [IQR, 6.5-11.5]). No patient required an additional postoperative scan due to neurological deterioration or clinical signs of shunt dysfunction.

All control patients underwent postoperative CT for LP shunts (median CTDIvol: 31.15 mGy [IQR, 23.5-34.2]). The volumetric dose index was significantly lower in the iCBCT group (p = 0.002). See Table 2 for radiation exposure.

**Table 2:** Radiation exposure.

variable	iCBCT group (n = 10)	control group (n = 10)
**volumetric dose index**		
(CBDIw/CTDIvol), mGy, median (IQR)	9.27 (6.5-11.5)	31.15 (23.5-34.2)

**modality-specific cumulative dose**		
DAP, mGy·cm^2^, median (IQR)	1894 (1573-2638)	n.a.
DLP, mGy·cm, median (IQR)	n.a.	616 (333-821)

conversion factor (k)		
head	0.065 mSv·mGy^−1^·cm^−2 14^	0.0021 mSv·mGy- (Khalil et al., 2024)·cm^−1 13^
abdomen	0.27 mSv·mGy^−1^·cm^−2 15^	0.015 mSv·mGy^−1^·cm^−1 13^

**effective dose** (mSv), median (IQR)		
intra-operative scan	3.4 (2.2-4.3)	n.a.
post-operative scan	n.a.	1.5 (1.0-4.8)

Abbr.: CBDIw, weighted cone-beam dose index; CTDIvol, volumetric computed tomography dose index; DAP, dose-area product; DLP, dose-length product.

**Fig. 2:**
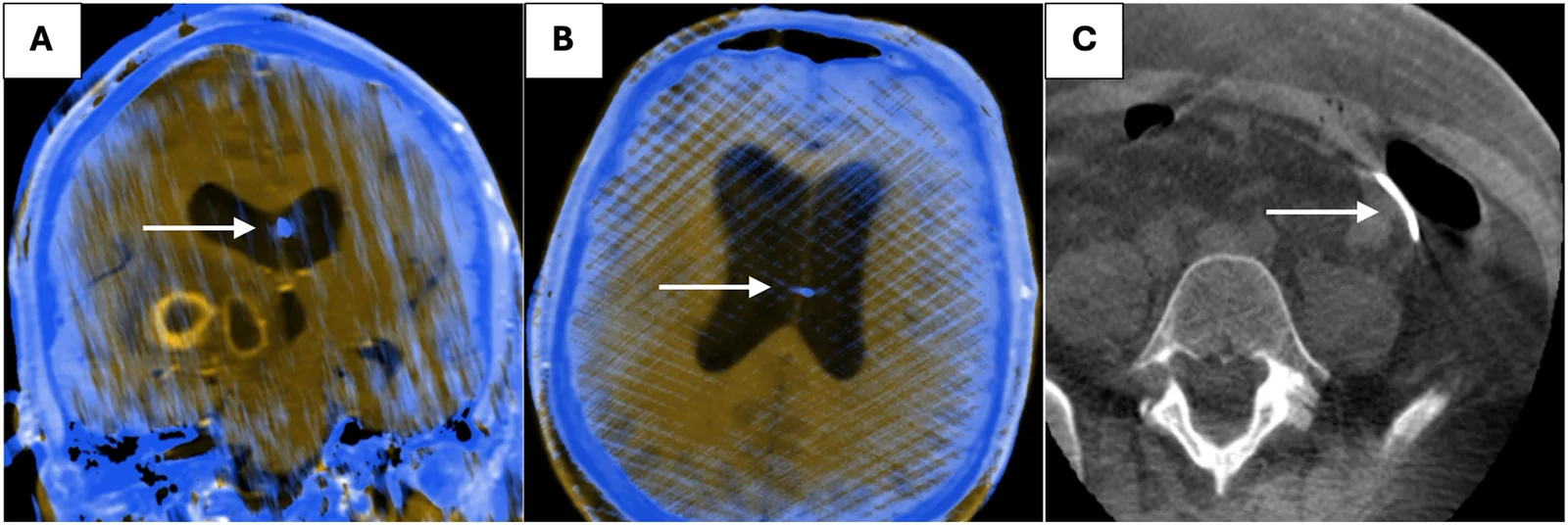
Intraoperative CBCT of the brain fused with preoperative MRI (A, coronar; B, axial) demonstrating cross-over positioning of the ventricular catheter, which prompted immediate intraoperative revision. A second scan confirmed correct intraventricular position of the catheter. Intraoperative CBCT of the abdomen (C) demonstrating intraperitoneal course and correct positioning of the distal catheter (white arrow).

Intraoperative CBCT detected cross-over positioning of the ventricular catheter in one VP shunt, which prompted immediate revision within the same surgical session (see Case 1 and Fig. 2). The repeat scan confirmed correct intraventricular catheter position. No further catheter malposition or kinking was detected intraoperatively, and no patient in either group required secondary revision surgery for malposition.

Surgical time was comparable between groups (median 65.5 min [IQR, 54–80] vs 60 min [IQR, 50–76]; p = 0.5901), whereas total OR time was longer in the iCBCT group due to image acquisition without statistical significance (median 142 min [IQR, 114–157] vs 127 min [IQR, 104–140]; p = 0.2948).

## 4 Case one

### 4.1 VP shunt implantation with iCBCT quality control

A 52-year-old patient was admitted on an emergency basis with progressive left-sided hemiparesis, aphasia, and psychomotor slowing. Neuroimaging revealed a large, heterogeneously contrast-enhancing lesion in the right basal ganglia with compression of the third ventricle and developing obstructive hydrocephalus. Following initial diagnostic surgery and ventriculocisternostomy, ventricular enlargement progressed, and VP shunt implantation was indicated. A right-sided VP shunt (proGAV 2.0 with ShuntAssistant, opening pressure 5 cmH_2_O and gravitational unit 30 cmH_2_O) was implanted. After final catheter placement, iCBCT was performed for quality control. Imaging revealed cross-over positioning of the ventricular catheter, with the catheter tip projecting into the contralateral ventricle. Immediate intraoperative revision was performed within the same surgical session, and a repeat iCBCT acquisition confirmed correct intraventricular catheter position. The distal catheter was appropriately positioned within the peritoneal cavity. Routine postoperative CT was omitted.

Radiation exposure amounted to a CBDIw of 11.76 mGy, a DAP of 3005 mGy·cm^2^ and an effective dose of 4.3 mSv for three intraoperative CBCT scans (two cranial scans + one abdominal scan)*.* Total skin-to-skin time was 54 min, with an overall operating room time of 81 min. The postoperative course was uneventful, and no shunt-related complications occurred (see Fig. 2).

## 5 Case two

### 5.1 LP shunt implantation with iCBCT quality control

An 80-year-old patient with suspected NPH underwent a lumbar tap test with reported clinical improvement. After discussion of treatment options including VP and LP shunting, LP shunt implantation was selected. A LP shunt system (Miethke M.blue valve, opening pressure 24 cmH_2_O, gravitational unit 5 cmH_2_O) was implanted with subcutaneous tunneling to the abdominal access site and intraperitoneal placement of the distal catheter. After final catheter placement, iCBCT was performed for quality control. Imaging confirmed correct positioning of both the lumbar and peritoneal catheter components on a single scan. No intraoperative revision was required, and routine postoperative imaging was omitted.

**Fig. 3:**
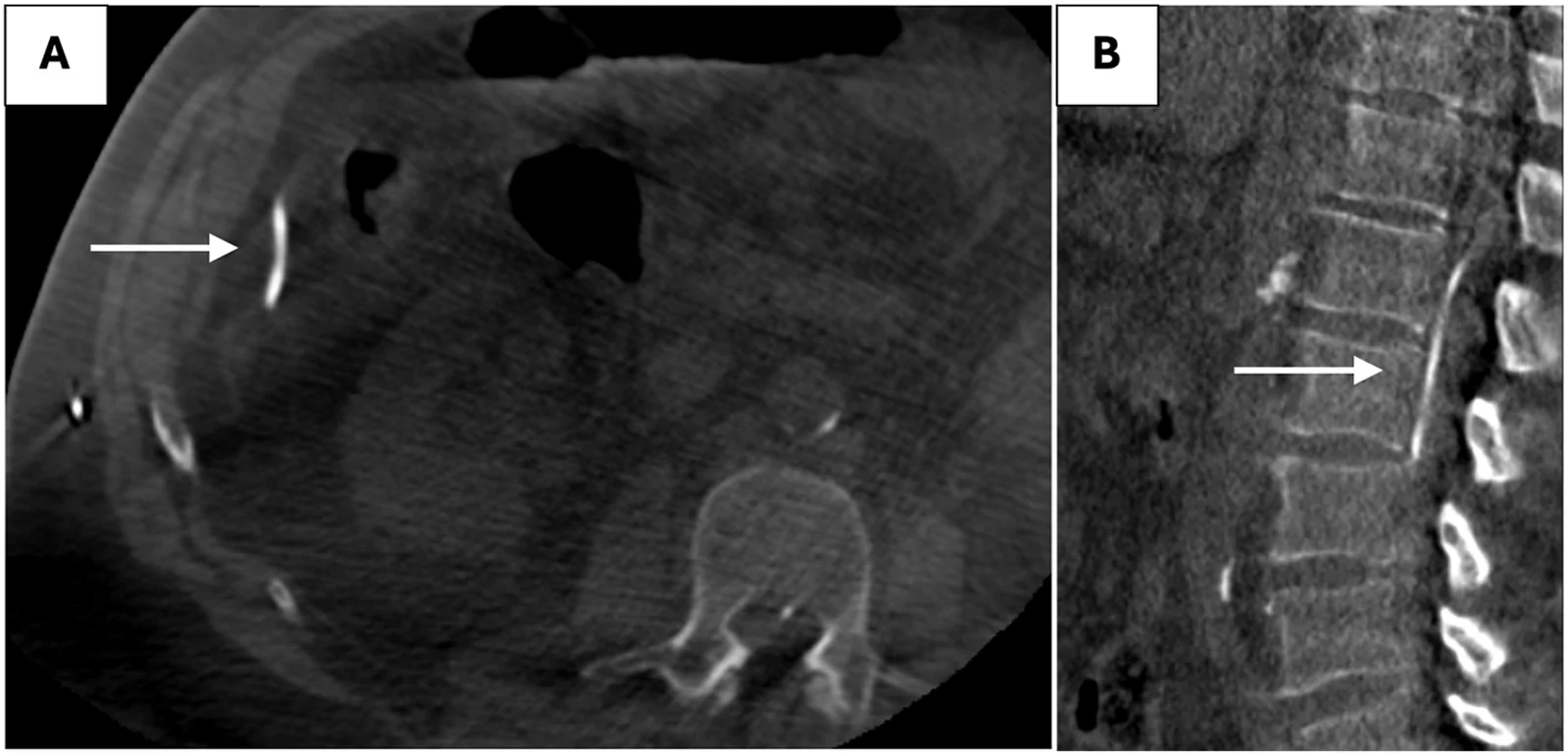
Intraoperative CBCT following LP shunt implantation, demonstrating correct intraspinal course of the lumbar catheter and intraperitoneal positioning of the abdominal catheter (white arrows) captured on a single scan: axial view (A) and sagittal reconstruction (B) of the same dataset.

Radiation exposure amounted to a CBDIw of 9.54 mGy, a DAP of 1605.8 mGy·cm^2^ and an effective dose of 3.4 mSv for iCBCT*.* Total skin-to-skin time was 55 min, with an overall operating room time of 120 min. The postoperative course was uneventful, and the patient was discharged after one day of inpatient observation (see Fig. 3).

## 6 Case three

### 6.1 VA shunt implantation with iCBCT quality control

A patient with hydrocephalus secondary to a cerebellar pilocytic astrocytoma, previously treated with a VP shunt, presented with suspected shunt dysfunction and severe abdominal pain due to multiple abdominal cysts attributed to the peritoneal catheter. Owing to persistent abdominal symptoms, the existing VP shunt was externalized, and conversion to a VA shunt was indicated. A VA shunt system (proGAV 2.0 valve, opening pressure 8 cmH_2_O, gravitational unit 20 cmH_2_O plus Mblue valve, opening pressure 5 cmH_2_O, gravitational unit 32 cmH_2_O) was implanted. After final catheter placement, iCBCT using the Loop-X system was performed for quality control. Imaging confirmed correct intraventricular and thoracic positioning of all shunt components. No intraoperative revision was required, and routine postoperative imaging was omitted.

**Fig. 4:**
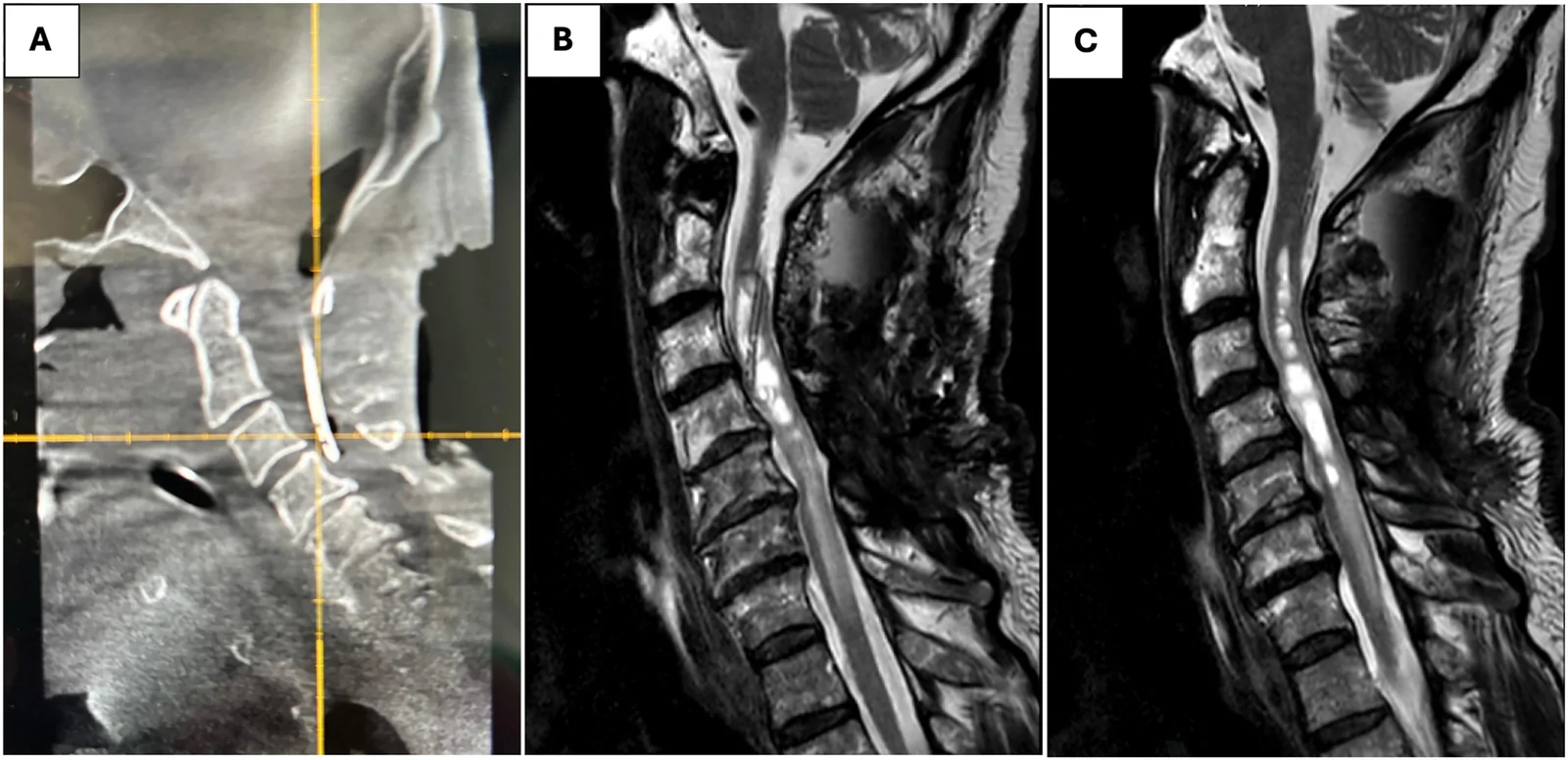
Intraoperative CBCT following VA shunt implantation. (A) Axial scan and (B) coronar reconstruction confirming correct distal catheter tip position within the right atrium following insertion via the internal jugular vein (white arrows), with no evidence of malposition, extravascular course, pneumothorax, or other intrathoracic complications. Intraoperative CBCT of the brain fused with preoperative MRI (C) demonstrating correct positioning of the ventricular catheter in the ipsilateral ventricle.

Radiation exposure amounted to a CBDIw of 4.81 mGy, a DAP of 1894.3 mGy·cm^2^ and an effective dose of 2.2 mSv for iCBCT*.* Total skin-to-skin time was 67 min, with an overall operating room time of 110 min. The postoperative course was uneventful, and no shunt-related complications occurred (see Fig. 4).

## 7 Case four

### 7.1 Cystodural shunt implantation with iCBCT quality control

A 63-year-old patient with a known cervical hemangioblastoma presented with progressive cystic enlargement of the cervical spinal cord. Due to progression of the cystic lesion, surgical fenestration and cyst fluid diversion were indicated. A cystosubdural shunt was implanted at the cervical level. Following hemilaminectomy at the C2/3 level and microsurgical dural opening, the cyst was accessed via a limited myelotomy. A catheter was inserted approximately 3 cm into the cystic cavity and subsequently positioned in the subarachnoid space with cranial drainage toward the cisterna magna. The catheter was secured to the arachnoid membrane. After final catheter placement, iCBCT was performed for quality control. Imaging confirmed correct cranial catheter course and appropriate positioning extending to the posterior margin of C4. No intraoperative revision was required. Postoperative MRI was performed to demonstrate the position of the catheter in relation to the cysts.

**Fig. 5:**
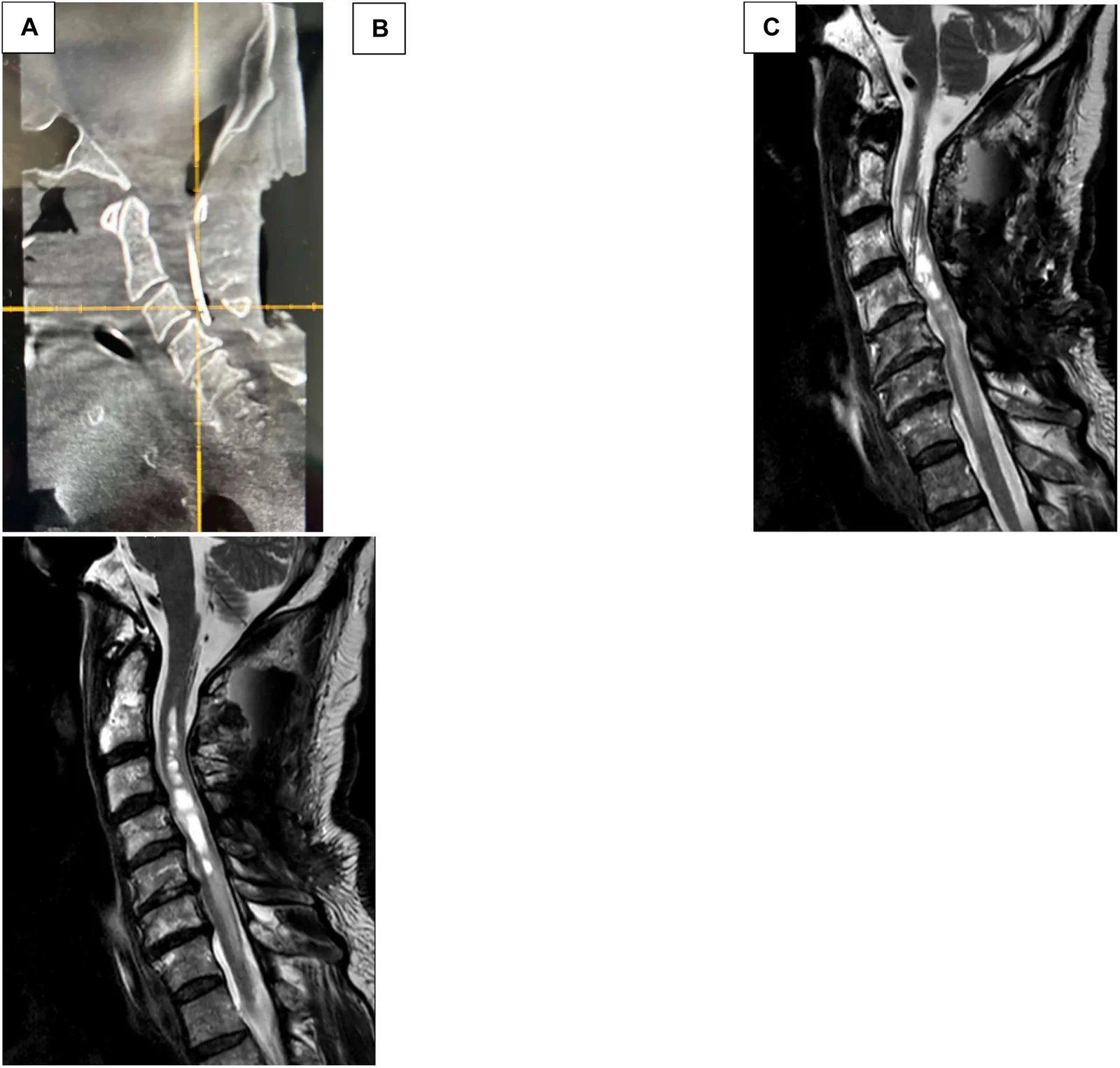
Intraoperative CBCT of the cervical spine confirming correct intraspinal positioning of the cyst shunt catheter following cyst fenestration (A). Postoperative MRI of the cervical spine confirming correct intracytic catheter positioning (B) and cranial diversion into the cisterna magna (C).

Radiation exposure amounted to a CBDIw of 6.89 mGy, a DAP of 2064.7 mGy·cm^2^ and an effective dose of 2.7 mSv for intraoperative CBCT. The postoperative course was uneventful, and no procedure-related complications occurred (see Fig. 5).

## 8 Discussion

In this prospective single-center study, an iCBCT-based workflow using a mobile CBCT platform enabled real-time, volumetric verification of the catheter position across the full spectrum of CSF diversion procedures. Intraoperative imaging detected cross-over positioning of the ventricular catheter in one VP shunt and enabled immediate correction within the same surgical session. Correct final catheter placement was confirmed intraoperatively in all patients, allowing omission of routine postoperative CT in all patients, while surgical time remained comparable to the conventional workflow. The iCBCT workflow has the potential to reduce radiation exposure and obviate the need for routine postoperative CT, which may reduce overall patient burden and hospital resources. By enabling intraoperative detection and correction of catheter malposition, iCBCT addresses a key limitation of conventional workflows, in which suboptimal catheter trajectories are identified only postoperatively and require secondary intervention under repeat anesthesia.

Radiation exposure represents an important consideration when implementing intraoperative imaging. Due to differences in dosimetric concepts, direct comparison between CBCT and diagnostic CT remains limited. In the present study, the volumetric dose index (CBDIw) was markedly lower in the iCBCT group than the CTDIvol of postoperative CT. However, the effective dose and the DAP was numerically higher in the iCBCT group, likely reflecting the larger combined anatomical volume captured in several iCBCT acquisitions. Furthermore, DAP (used for iCBCT) and DLP (used for diagnostic CT) are physically distinct quantities that require different conversion factors. Effective doses derived from these two metrics are therefore not directly comparable, even when expressed in the same unit. This apparent discrepancy between dose index and effective dose is consistent with an independent dosimetric comparison of the same CBCT system (Loop-X) against conventional CT, which found that manufacturer-reported dose indices (CBDI vs. CTDI) overestimated the true dose difference by nearly sixfold, whereas the DLP was comparable between modalities (Larsson et al., 2026). A lower dose index per acquisition therefore does not necessarily translate into a lower cumulative radiation burden, depending on acquisition protocol and scan coverage. Rather than a uniform reduction in radiation exposure, the principal advantage of the proposed workflow lies in replacing rather than supplementing postoperative CT, avoiding an additional imaging examination and its associated logistical burden.

The four illustrative cases highlight scenarios in which this advantage is particularly relevant: intraoperative detection of suboptimal catheter tip position (Case 1), single-acquisition assessment of intraspinal and intraperitoneal catheter components in LP shunting (Case 2), confirmation of atrial-tip position without intraprocedural transesophageal echocardiography in VA shunting (Case 3), and immediate verification of intraspinal trajectory in a complex cyst diverting shunt (Case 4).

Operative efficiency was preserved: both surgical and total OR time did not differ significantly between groups, although total OR time was numerically longer in the iCBCT group due to the additional imaging step. The additional time required for image acquisition must be balanced against the logistical effort associated with postoperative CT, including patient transport, resource utilization, and potential delays in postoperative discharge. In this context, intraoperative imaging may contribute to a more streamlined and patient-centered pathway.

A notable aspect of this study is the applicability of iCBCT across different types of CSF diversion procedures, including VP, LP, VA, and even complex cyst shunts – however, the cohort was dominated by LP shunts (6 of 10 patients per group), with only a single VP and a single VA shunt. While previous studies have predominantly focused on ventricular catheter placement, these individual cases suggest that iCBCT provides sufficient image quality to assess both proximal and distal catheter components across multiple anatomical compartments. Given the limited representation of VP and VA shunts, this observation should be regarded as a preliminary, case-level feasibility finding rather than a conclusion generalizable across shunt types.

Several limitations should be acknowledged. The sample size is small, and the study is exploratory in nature, limiting statistical power and precluding definitive conclusions regarding revision rates or shunt survival. Although the observed reduction in radiation exposure was statistically significant, this result should likewise be regarded as preliminary given the small interim sample size. Furthermore, radiation dose assessment is based on system-derived metrics, and differences between CBCT and CT dosimetry restrict direct comparability.

## 9 Conclusion

Intraoperative CBCT enables reliable real-time verification of catheter placement in CSF shunt surgery and can be integrated into the surgical workflow without prolonging operative time. By allowing omission of routine postoperative CT, this approach has the potential to reduce patient burden and optimize perioperative care. Across VP, LP, VA, and complex cyst-shunt configurations, intraoperative quality control was feasible and enabled omission of a separate postoperative imaging examination, with a lower per-acquisition dose index than conventional CT, although cumulative effective dose was not uniformly lower. Larger, multicenter prospective studies will be required to define the impact of iCBCT-guided shunt implantation on revision rates, shunt survival, and health-economic outcomes and to confirm the magnitude of the radiation-dose reduction observed here.

## Consent to participate

The requirement for informed consent was waived by the Institutional Review Board of Goethe University Frankfurt due to the retrospective nature of the study.

## Consent to publish

Not applicable. The manuscript does not contain any individual person's identifiable data, images, or videos.

## Ethics approval

The study protocol was reviewed and approved by the Ethics Committee of the Faculty of Medicine, Goethe University Frankfurt, Germany (No. 2025-2373) and was conducted in accordance with the Declaration of Helsinki and applicable regulatory requirements. The study was prospectively registered in the German Clinical Trials Register (Deutsches Register Klinischer Studien, DRKS; trial identifier DRKS00037762). Written informed consent was obtained from all patients in the prospective cohort. For the retrospective cohort, the requirement for informed consent was waived by the Ethics Committee of Goethe University Frankfurt am Main, Germany.

## Author contributions

Conception and design: Marcus Czabanka, Lina-Elisabeth Qasem, Vincent Prinz.

Acquisition of data: Lina-Elisabeth Qasem, Dilara Karakaya, Christian Seemann, Nadine Angelika Döpke, Leonhard Mann, Fee Keil, Fatma Kilinç, Paul Kendlbacher.

Analysis and interpretation of data: Lina-Elisabeth Qasem.

Drafting the manuscript: Lina-Elisabeth Qasem, Vincent Prinz.

All authors reviewed the results and approved the final version of the manuscript.

## Data availability

The datasets generated and analyzed during the current study are not publicly available due to data-protection regulations but are available from the corresponding author on reasonable request.

## Declaration of generative AI in scientific writing

During the preparation of this work, the authors used a large language model-based assistant (Claude) for language editing and to improve readability of the manuscript. After using this tool, the authors reviewed and edited the content as needed and take full responsibility for the content of the publication.

## Funding

The authors declare that no funds, grants, or other support were received during the preparation of this manuscript.

## Competing interests

The authors have no relevant financial or non-financial interests to disclose.
