# Supplementary material for: Intraoperative cone-beam CT for real-time quality control in CSF shunt surgery: a prospective workflow study
Source: Brain Spine. 2026 Aug 18;6:106269. doi: 10.1016/j.bas.2026.106269 (PMC13521095; doi:10.1016/j.bas.2026.106269)
Supplement: Supplementary file 1 [file mmc1.docx]

**STROBE Statement — Checklist of items that should be included in reports of cohort studies**

Manuscript: **Intraoperative cone-beam CT for real-time quality control in CSF shunt surgery: a prospective workflow study**

| Section | Item No. | Recommendation | Reported on / Comment |
| --- | --- | --- | --- |
| Title and abstract | 1 | (a) Indicate the study's design with a commonly used term in the title or the abstract | Title; Abstract – Material and methods |
|  |  | (b) Provide in the abstract an informative and balanced summary of what was done and what was found | Abstract |
| Introduction |  |  |  |
| Background/rationale | 2 | Explain the scientific background and rationale for the investigation being reported | Introduction, paragraphs 1–3 |
| Objectives | 3 | State specific objectives, including any prespecified hypotheses | Introduction, last paragraph; Methods – Research question/Endpoints |
| Methods |  |  |  |
| Study design | 4 | Present key elements of study design early in the paper | Methods – Study design |
| Setting | 5 | Describe the setting, locations, and relevant dates, including periods of recruitment, exposure, follow-up, and data collection | Methods – Study design (Department of Neurosurgery, Goethe University Frankfurt am Main; January–April 2026 and September–December 2025) |
| Participants | 6 | (a) Give the eligibility criteria, and the sources and methods of selection of participants. Describe methods of follow-up | Methods – Study design; Ethics |
|  |  | (b) For matched studies, give matching criteria and number of exposed and unexposed | Methods – Study design (1:1 matched by shunt type, diagnosis, sex, age; 10 vs 10) |
| Variables | 7 | Clearly define all outcomes, exposures, predictors, potential confounders, and effect modifiers. Give diagnostic criteria, if applicable | Methods – Study design (primary/secondary endpoints); Technical note |
| Data sources/measurement | 8 | For each variable of interest, give sources of data and details of methods of assessment (measurement). Describe comparability of assessment methods if there is more than one group | Methods – Technical note (CBDIw / CTDIvol); Statistical analysis |
| Bias | 9 | Describe any efforts to address potential sources of bias | Methods – Study design (matched cohort, standardized acquisition protocol) |
| Study size | 10 | Explain how the study size was arrived at | Methods – Statistical analysis (exploratory, no formal sample-size calculation) |
| Quantitative variables | 11 | Explain how quantitative variables were handled in the analyses. If applicable, describe which groupings were chosen and why | Methods – Statistical analysis |
| Statistical methods | 12 | (a) Describe all statistical methods, including those used to control for confounding | Methods – Statistical analysis |
|  |  | (b) Describe any methods used to examine subgroups and interactions | Not applicable (exploratory descriptive study) |
|  |  | (c) Explain how missing data were addressed | Not applicable (no missing data for primary endpoint) |
|  |  | (d) If applicable, explain how loss to follow-up was addressed | Not applicable (in-hospital observational endpoints) |
|  |  | (e) Describe any sensitivity analyses | Not applicable |
| Results |  |  |  |
| Participants | 13 | (a) Report numbers of individuals at each stage of study – eg numbers potentially eligible, examined for eligibility, confirmed eligible, included in the study, completing follow-up, and analysed | Results – Baseline characteristics |
|  |  | (b) Give reasons for non-participation at each stage | Not applicable (consecutive enrolment) |
|  |  | (c) Consider use of a flow diagram | Not applicable (small consecutive cohort) |
| Descriptive data | 14 | (a) Give characteristics of study participants (eg demographic, clinical, social) and information on exposures and potential confounders | Results – Baseline characteristics; Table 1 |
|  |  | (b) Indicate number of participants with missing data for each variable of interest | No missing data |
|  |  | (c) Summarise follow-up time (if applicable) | Not applicable (in-hospital follow-up) |
| Outcome data | 15 | Report numbers of outcome events or summary measures over time | Results – Radiological and surgical parameters; Cases 1–4 |
| Main results | 16 | (a) Give unadjusted estimates and, if applicable, confounder-adjusted estimates and their precision | Results – Radiological and surgical parameters |
|  |  | (b) Report category boundaries when continuous variables were categorized | Not applicable |
|  |  | (c) If relevant, consider translating estimates of relative risk into absolute risk for a meaningful time period | Not applicable |
| Other analyses | 17 | Report other analyses done – eg analyses of subgroups and interactions, and sensitivity analyses | Not applicable |
| Discussion |  |  |  |
| Key results | 18 | Summarise key results with reference to study objectives | Discussion, paragraph 1 |
| Limitations | 19 | Discuss limitations of the study, taking into account sources of potential bias or imprecision. Discuss both direction and magnitude of any potential bias | Discussion – Limitations |
| Interpretation | 20 | Give a cautious overall interpretation of results considering objectives, limitations, multiplicity of analyses, results from similar studies, and other relevant evidence | Discussion, paragraphs 2–4 |
| Generalisability | 21 | Discuss the generalisability (external validity) of the study results | Discussion – Limitations; Conclusion |
| Other information |  |  |  |
| Funding | 22 | Give the source of funding and the role of the funders for the present study and, if applicable, for the original study on which the present article is based | Declarations – Funding |

*Note: An Explanation and Elaboration article discusses each checklist item and gives methodological background and published examples of transparent reporting. The STROBE checklist is best used in conjunction with this article (freely available on the websites of PLoS Medicine, Annals of Internal Medicine, and Epidemiology). Information on the STROBE Initiative is available at http://www.strobe-statement.org.*
